# Transcriptome analysis and identification of genes associated with leaf crude protein content in foxtail millet [*Setaria italica* (L.) P. Beauv.]

**DOI:** 10.3389/fgene.2023.1122212

**Published:** 2023-01-20

**Authors:** Yanjiao Cui, Dan Liu, Zilong Zhao, Jing Zhang, Suying Li, Zhengli Liu

**Affiliations:** ^1^ Department of Life Sciences, Tangshan Normal University, Tangshan, China; ^2^ Tianjin Key Laboratory of Crop Genetics and Breeding, Institute of Crop Sciences, Tianjin Academy of Agricultural Sciences, Tianjin, China

**Keywords:** foxtail millet, Chinese chestnut, spruce spider mite, crude protein content, transcriptome analysis

## Abstract

**Introduction:** Spruce spider mite is a primary insect pest of Chinese chestnut in China and seriously influences its yield and quality. However, the current management against this mite is costly and poorly effective. In previous research, we bred several foxtail millet materials for interplanting with chestnut tree, and found that they had high levels of crude protein (CP) in leaves and attracted spruce spider mite to feed on the leaves, thereby reducing chestnut damage.

**Methods:** In this study, four foxtail millet varieties with significant differences in leaf crude protein content were used for high-throughput sequencing and identification of genes associated with leaf crude protein content. Gene enrichment analyses were carried out to comprehend the functions of these genes and the biological processes in which they are involved. In addition, transcription factors (TFs) were evaluated.

**Results:** 435 differentially expressed genes (DEGs) were identified, suggesting their potential role in crude protein accumulation. Some differentially expressed genes were found to be associated with nitrogen metabolism and ubiquitin-mediated proteolysis pathways. Moreover, we identified 40 TF genes categorized into 11 transcription factor families.

**Discussion:** Our findings represent an important resource that clarifies the mechanisms of accumulation and control of leaf crude protein in foxtail millet, and provide an opportunity for suppression of spruce spider mite attack on Chinese chestnut by interplanting with foxtail millet varieties with high concentrations of leaf crude protein.

## Introduction

Chestnut has been cultivated in China for more than 2700 years (Adua, 1999). It is an economically valuable plant, its wood used for timber, its leaf and flower utilized as raw material in the pharmaceutical and cosmetic industries, and its fruit peel a source of extracted tannin. Most of all, its fruit is a popular food resource with high nutritional value. The genus *Castanea* comprises 12 world chestnut species, including four cultivated species: Chinese chestnut (*Castanea mollissima* Blume), Japanese chestnut (*Castanea crenata* Sieb. et Zucc.), American chestnut (*Castanea dentata* [Marsh.] Borkh), and European chestnut (*Castanea sativa* Miller) (Jiang et al., 2019). Among these, Chinese chestnut and Japanese chestnut are the most commercially important (Tanaka and Kotobuki, 1992).

Chinese chestnut is commonly grown in China for its fruits. However, several insect pests can seriously damage the tree’s trunk, buds, and fruit, and reduce nut production and quality (Gokce et al., 2020). One such pest is the spruce spider mite (*Oligonychus ununguis* Jacobi), a typical polyphagous mite that consumes the chestnut’s leaves (Wang et al., 1989; Wang et al., 2010). In the main chestnut-producing region of China, the occurrence of spruce spider mite generally leads to a 15%–50% decline in production (Wang et al., 1991).

To date, several biological and chemical control measures have been taken to prevent this mite from attacking trees in China, including the use of natural enemy species and pesticides (Wang et al., 2010; Zhao and Fan, 2019). Nevertheless, because Chinese chestnut trees are tall and mostly distributed in mountains, it is inconvenient to spray pesticides. Moreover, it often results in the development of resistance to pesticides and environmental pollution, thereby slowing the movement toward green, pollution-free production of crops and restricting the development of the Chinese chestnut industry. In sum, the control measures for spruce spider mite prevention are currently expensive and poorly effective.

In previous work, we developed a number of foxtail millet (*Setaria italica* (L.) P. Beauv.) materials for interplanting with chestnut. They were early-maturing, shade-tolerant, could thrive beneath Chinese chestnut trees, realizing the high-efficient utilization of land resources under the trees. Surprisingly, we discovered that the leaves of these materials were attacked by spruce spider mites. This decreased the rate of bad chestnut nuts resulting from mite attack by 5%–15%, while damage to the millet was minimal (Li et al., 2022). Furthermore, these materials had a high content of crude protein (CP) in their leaves.

Plant–insect interactions are intricate and dynamic. To overcome insect attack, plants develop different defense mechanisms, including physical and chemical defenses (War et al., 2012; Belete, 2018). The first mechanical barrier to feeding is formed by structural features on the leave surface, such as a waxy cuticle, spines and thorns, trichomes, and thick and lignified cell walls. Secondary metabolites, including phenolics, flavonoids, tannins, and lectins, provide the next barriers of protection. Among chemical constituents, nutrients such as amino acids, vitamins, proteins, and carbohydrates have been also reported to affect the relationship between plants and insects (Li and Jin, 2005; Yang et al., 2014; Li and Li, 2018). In one study, nymphal density and the leaf damage index of grape leafhopper (*Erythroneura apicalis* Nawa) were negatively correlated with CP levels in grape leaf (Fan et al., 2008). Another study reported that total soluble protein content in cotton leaves was positively associated with the population densities of thrip (*Thrips tabaci*), whitefly (*Bemesia tabaci*), and jassid (*Amrasca devastans*), and these pests were more prone to attack cotton genotypes with high concentrations of such proteins (Rizwan et al., 2021). Hence, the protein content of host plant leaves may be related to the degree of insect infestation, which could explain the effect of the foxtail millet materials we bred in previous work. Consequently, it is important to mine genes related to CP accumulation in foxtail millet leaves and to elucidate their regulation mechanisms. This would be helpful for breeding of foxtail millet varieties with high concentrations of leaf CP, and provide an effective, economically, and environmentally safe control measure for spruce spider mite through interplanting.

In this study, we performed transcriptome analyses on four foxtail millet varieties with significantly different leaf CP levels to identify genes associated with leaf CP content. We identified and characterized differentially expressed genes (DEGs) and conducted gene enrichment analyses to investigate their possible functions. We also predicted differentially expressed transcription factor (TF) genes. Our results lay a foundation for clarifying the biological processes and regulation mechanisms underlying leaf CP accumulation, and provide potentially precious gene resources for the future development of foxtail millet varieties with high concentrations of leaf CP.

## Materials and methods

Below we provide a summary of our methods. More details can be found in the Supplementary Materials and Methods.

### Plant materials

Four hybrid varieties of foxtail millet (51950, 12950, 1121, 57295) and a conventional variety (JG32) grown in an experimental field of Beiguan Village, Tangshan, China (40°10'N, 118°28'E) were utilized. The millet was sown in 10 m long rows with 0.5 m between rows. For each variety, three standard plants were chosen, and the middle parts of the first, second, and third leaves from the top of each were collected during the booting stage. Then we mixed and ground the sampled leaves for CP content analysis, RNA-seq analysis, and quantitative real-time PCR (qRT-PCR) validation.

### Chemical analysis of crude protein concentration

Hebei Jintianfeng Grain Trading Co., Ltd. (China) conducted the CP concentration analysis. Total nitrogen content was obtained using the Kjeldahl method (Kjeldahl, 1883), and the value was multiplied by 6.25 to calculate CP content. Each sample had three biological replicates.

To screen varieties with different CP levels, JG32 was used as a control, which has been found to be suitable for use in interplanting with Chinese chestnut. Three varieties with high CP concentrations (51950, 12950, and 1121) and one with a low concentration (57295) were selected for high-throughput sequencing. The CP levels are listed in Supplementary Table S1.

### Library construction, high-throughput sequencing, and transcriptome analysis

To mine genes associated with leaf CP content, the leaves of each variety were sampled as mentioned above. RNA extraction, library construction, high-throughput sequencing, and transcriptome assembly were carried out as described previously (Li et al., 2022).

### Screening of differentially expressed genes

Fragments per kilobase of transcript per million fragments mapped (FPKM) values were used to determine the gene expression levels (Trapnell et al., 2010). To detect DEGs, the software edgeR version 3.3.3 (Robinson et al., 2009) was used, and genes with a |log_2_ (fold change)| value >1 and adjusted *p*-value (padj) < 0.05 were considered significant DEGs.

### qRT-PCR validation

The qRT-PCR validation was conducted as described previously (Liu et al., 2021). Three independent replicates were analyzed, and the primers used are listed in Supplementary Table S2.

### Functional enrichment analyses

GO functional enrichment analysis and KEGG pathway analysis of DEGs were conducted using topGO (Yu et al., 2010) and KOBAS 3.0 (Xie et al., 2011), respectively.

### Identification of TFs

The online webserver PlantTFDB 5.0 (http://planttfdb.gao-lab.org/prediction.php) with default parameters was used to identify TF families (Jin et al., 2017). In this tool, ESTScan 3.0 was employed to analyze coding regions in the provided input sequences (Iseli et al., 1999). A heatmap of differentially expressed TF genes was generated using TBtools software (Chen et al., 2020).

## Results

### Transcriptome sequencing and assembly

To characterize genes associated with biosynthesis and accumulation of leaf CP, 12 cDNA libraries from leaf samples of the four varieties with different CP levels were used for RNA sequencing (three library repeats for each variety). Approximately 645.17 M raw reads were generated using an Illumina Novaseq PE150 platform. After quality control, we obtained 641.29 M clean reads and found that 94.76%–95.65% were mapped to the foxtail millet reference genome (Setaria_italica_v2.0, http://plants.ensembl.org/Setaria_italica/Info/Index), provided in a previous report (Liu et al., 2022). Furthermore, Pearson’s correlation coefficients between biological replicates showed that the biological replicates were highly correlated (Liu et al., 2022), indicating the reliability of the RNA-seq results for gene expression analysis. In total, 30,141 genes were discovered. The FPKM values for each gene were computed and are listed in Supplementary Table S3.

### Identification of differentially expressed genes

**FIGURE 1:**
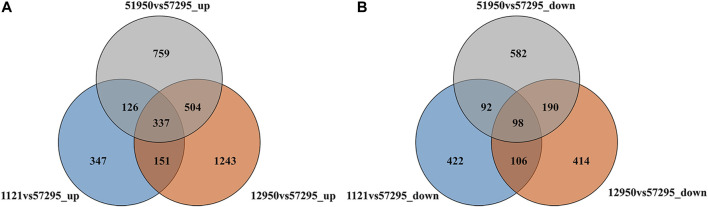
Venn map depicting the number of DEGs between foxtail millet varieties with high and low leaf crude protein content. **(A)** and **(B)** show the distribution of upregulated and downregulated DEGs in each comparison, respectively.

As shown in Figure 1, compared to the low-CP variety 57295, 1726, 2235, and 961 DEGs were upregulated in the high-CP varieties 51950, 12950, and 1121, respectively. In all, 3467 upregulated DEGs were identified, 337 of which were shared among the three high-CP varieties. Likewise, 962, 808, and 718 DEGs were downregulated in 51950, 12950, and 1121, respectively. In all, 1904 downregulated genes were identified, among which 98 were shared. Differential expression analysis provided 435 common DEGs shared in all comparisons, representing possible candidate genes associated with CP accumulation in foxtail millet.

### Validation of differentially expressed genes using qRT-PCR

**FIGURE 2:**
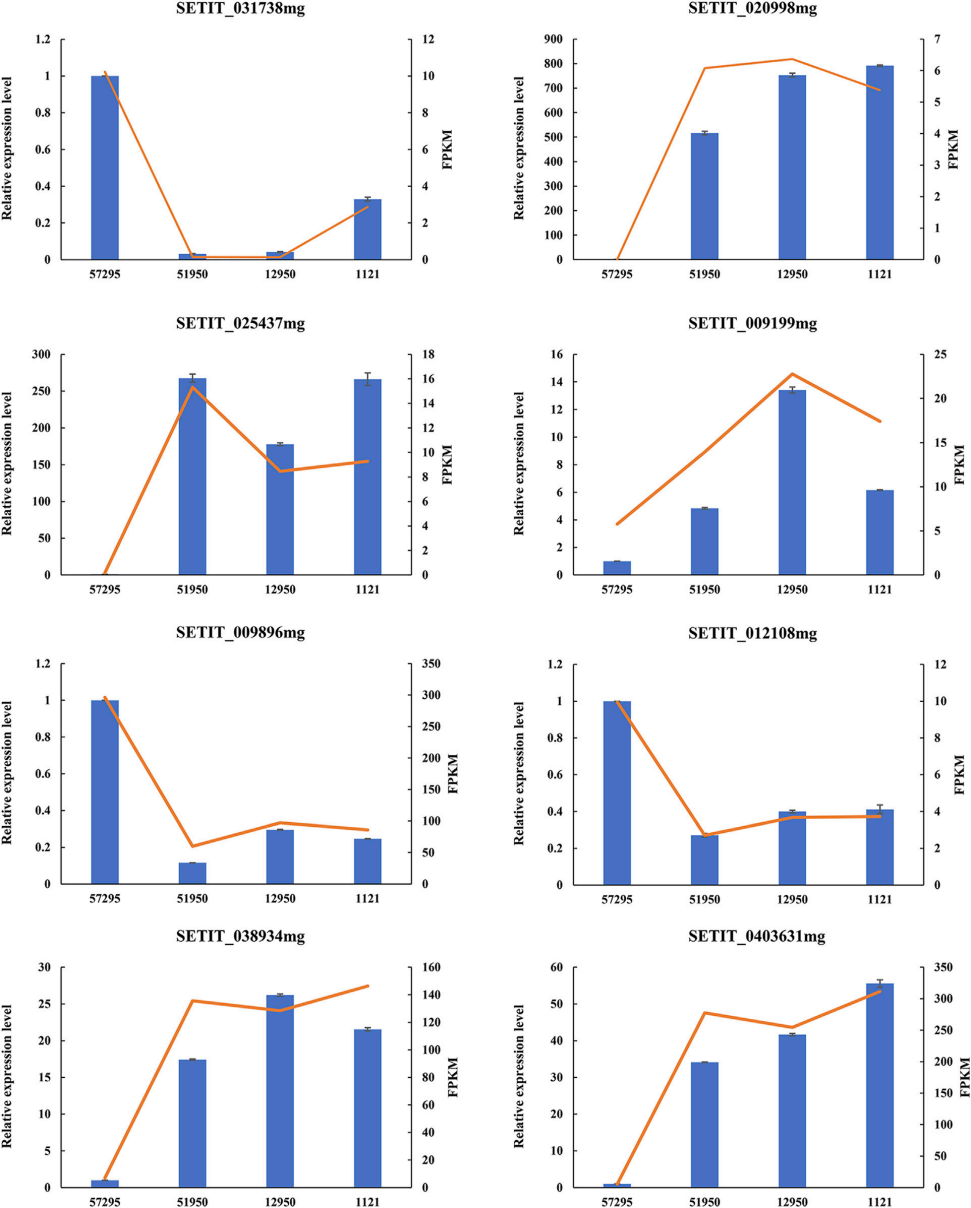
Validation of eight DEGs by qRT-PCR. The *X*-axis represents foxtail millet varieties with different levels of leaf crude protein content, and the *Y*-axis (left side) indicates the relative expression level of selected genes determined by qRT-PCR (blue columns). The *SiACTIN* gene was used as an internal control, and the transcript level of genes in variety 57295 was set as 1.0. Error bars represent standard error (*n* = 3). The *Y*-axis (right side) depicts the expression level of genes in RNA-seq data (evaluated by FPKM, red lines).

To verify the accuracy of the RNA-seq data, the relative expression levels of eight randomly selected DEGs were examined *via* qRT-PCR. These eight DEGs included five genes that were predicted to be upregulated in varieties with high CP concentrations (SETIT_020998mg, SETIT_025437mg, SETIT_009199mg, SETIT_038934mg, SETIT_0403631 mg), and three genes predicted to be downregulated (SETIT_031738mg, SETIT_009896mg, SETIT_012108 mg). As shown in Figure 2, the expression profiles were in line with the RNA-seq data, further demonstrating the reliability of our RNA-seq data.

### Gene enrichment analysis of differentially expressed genes

**FIGURE 3:**
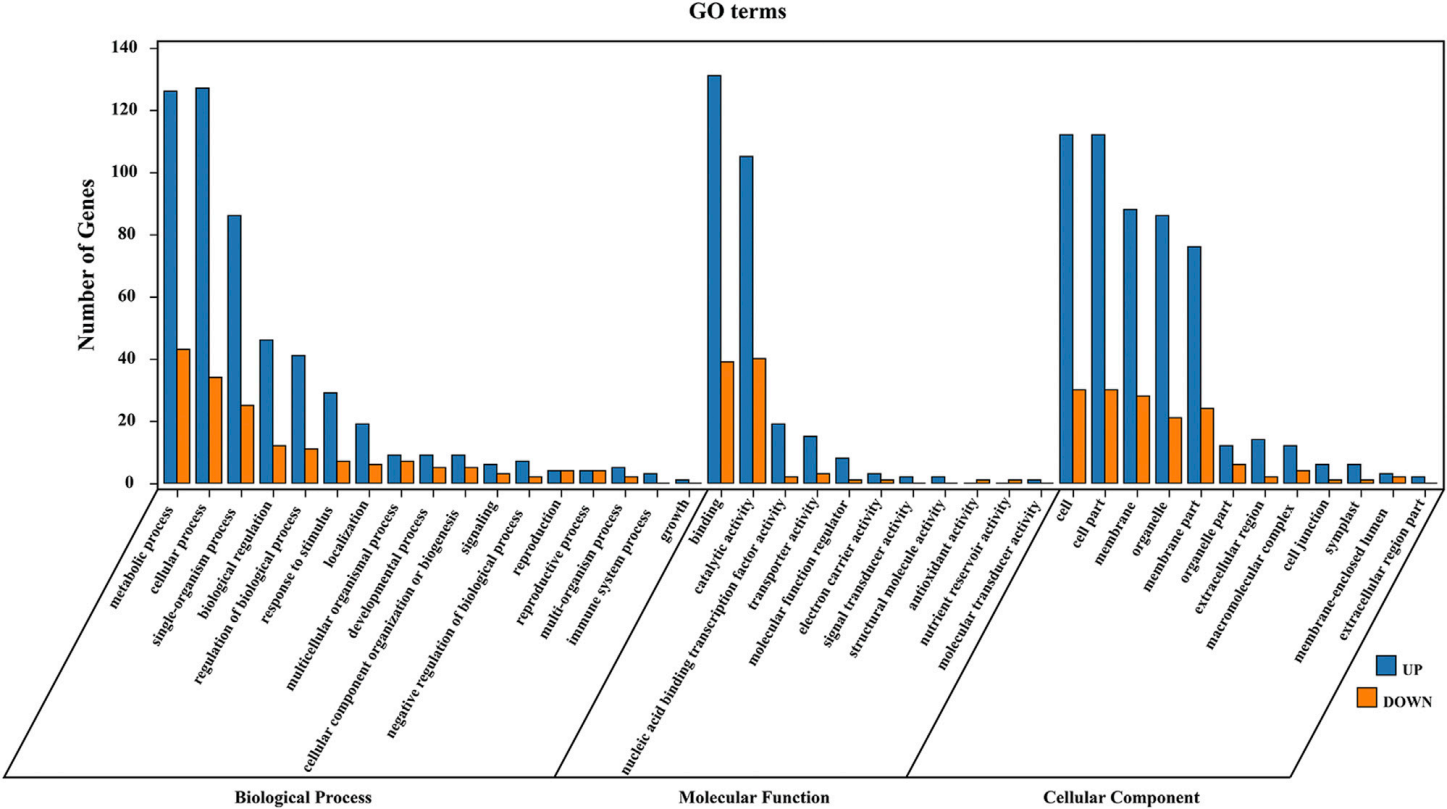
GO enrichment analysis of DEGs. Enriched GO terms of biological processes, molecular function, and cellular components are shown. Blue represents upregulated DEGs in varieties with high leaf crude protein content, and red represents downregulated DEGs.

For 435 common DEGs in all comparisons, GO category enrichment analysis was conducted using topGO (Yu et al., 2010) to determine their potential biological processes and functions (Supplementary Table S4). These DEGs were annotated to three categories and 40 GO terms: 42.5% of the terms were classified as biological processes, 27.5% were molecular functions, and 30% were cellular components (Figure 3). In the biological process category, genes were most enriched in metabolic processes (GO:0008152) and cellular processes (GO:0009987). In the molecular function category, most genes fell into the binding (GO:0005488) and catalytic activity (GO:0003824) subgroups. In the cellular component category, the genes mainly belonged to cell (GO:0005623) and cell part (GO:0044464) subgroups.

**FIGURE 4:**
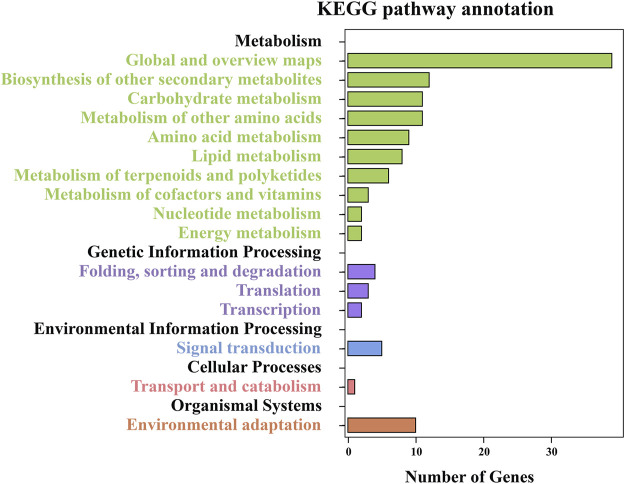
KEGG pathway analysis of enriched DEGs. The numbers of DEGs are shown on the *X*-axis, and the KEGG pathway terms are shown on the *Y*-axis.

To further understand the functional categorizations, the 435 DEGs were also subjected to KEGG enrichment analysis (Table S5); 174 DEGs were assigned to 60 enrichment pathways categories, of which the top five were metabolic pathways (35 genes, 20.11%), biosynthesis of secondary metabolites (28, 16.09%), plant–pathogen interactions (8, 4.60%), cyanoamino acid metabolism (7, 4.02%), and phenylpropanoid biosynthesis (7, 4.02%). These pathways were classified into five main categories and 16 subcategories, with the top five subcategories being global and overview maps, biosynthesis of other secondary metabolites, carbohydrate metabolism, metabolism of other amino acids, and environmental adaptation (Figure 4). The enrichment analysis illustrated that changes in leaf CP content significantly impacted the life processes of foxtail millet.

Amino acids are necessary for the formation of proteins and other related compounds, with nitrogen being the essential constituent (Mokhele et al., 2012). Due to the close relationship between nitrogen and protein and other nitrogenous compounds, nitrogen metabolism in plants directly affects CP content. According to the KEGG pathway analysis, the genes SETIT_014128 mg and SETIT_014124 mg present in carbonic anhydrase (EC:4.2.1.1) were enriched in the nitrogen metabolism pathway and both were upregulated in leaf tissues of foxtail millet varieties with high CP concentrations.

As one of the most prevalent protein posttranslational modifiers, ubiquitin (Ub) is a small peptide of 76 amino acids that can be attached to target proteins *via* ubiquitination (Hershko et al., 1998). Protein ubiquitination is involved in the regulation of proteolysis, subcellular localization, and the stability and activity of substrate proteins (Zhou et al., 2017). As per the KEGG analysis, two genes, SETIT_004756 mg present in E3 ubiquitin-protein ligase SIAH1 (EC:2.3.2.27) and SETIT_025290 mg present in ubiquitin-conjugating enzyme E2 J2 (EC:2.3.2.23), were annotated to ubiquitin-mediated proteolysis, a major pathway of degradation of cellular proteins. Based on differential expression analysis, SETIT_004756 mg and SETIT_025290 mg were downregulated and upregulated in varieties with high CP concentrations, respectively.

### TF analysis

**FIGURE 5:**
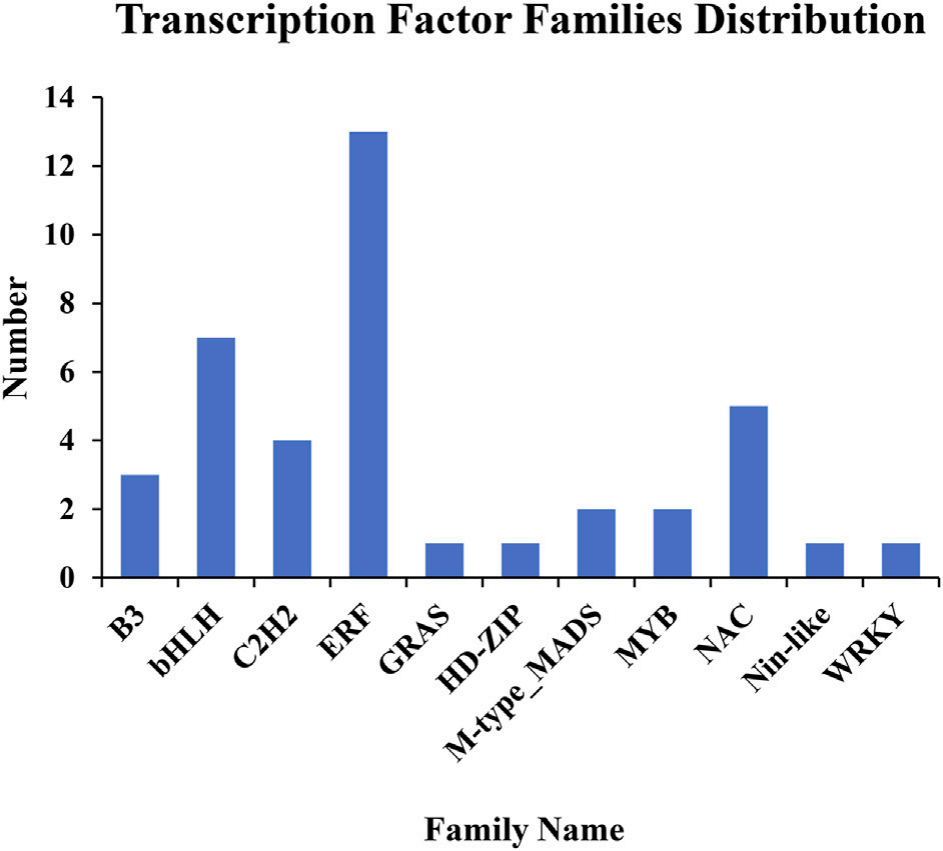
Graphical distribution of transcription factor families enriched in DEGs.

**FIGURE 6:**
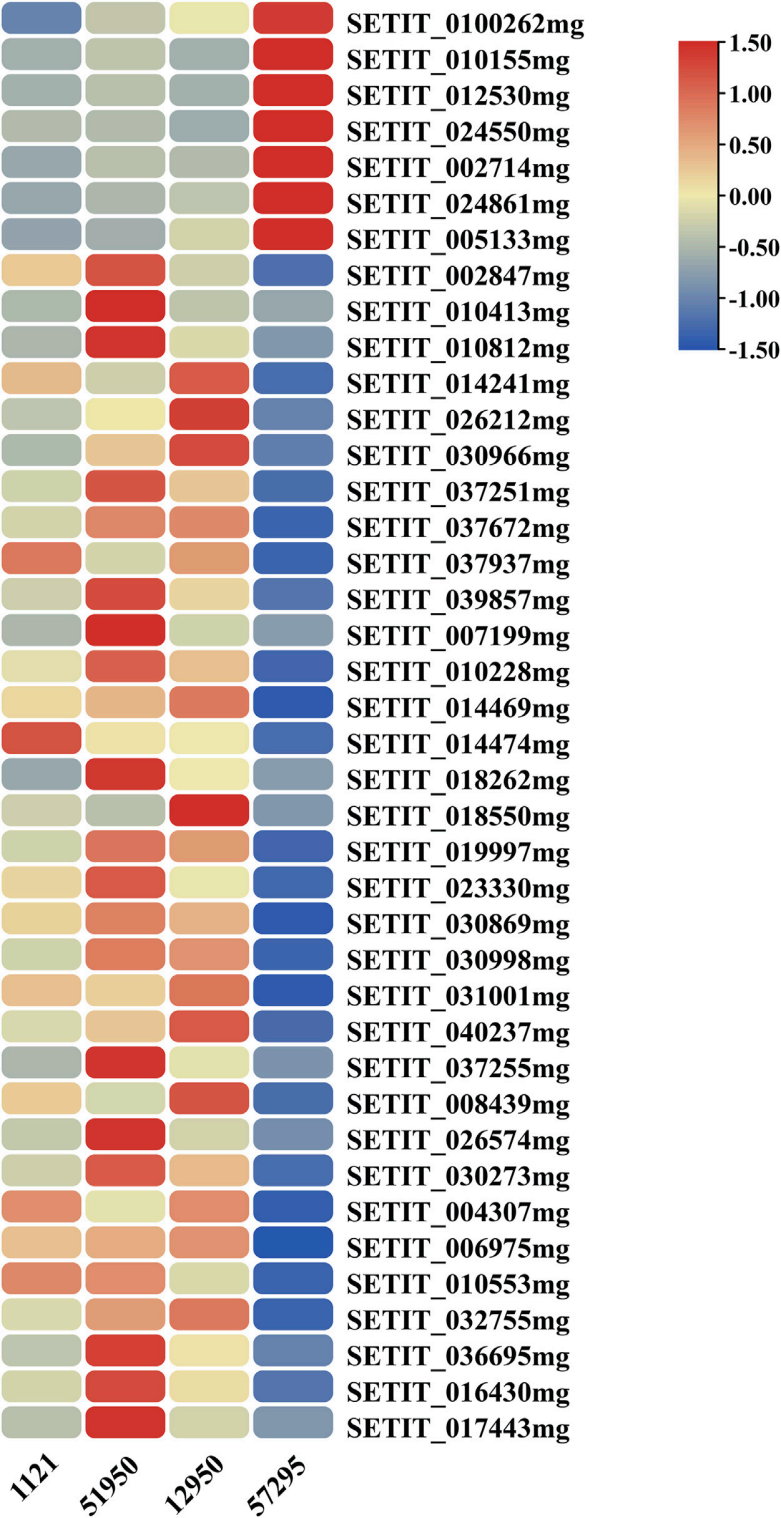
Heatmap of the differentially expressed transcription factor genes. The color gradient indicates the FPKM value of genes; red and blue represent high and low expression, respectively.

The GO enrichment analysis revealed that several DEGs may function in regulation of transcription and have TF activity (Supplementary Table S4). TFs are DNA-binding proteins that mediate many processes by playing a crucial role in gene transcription and expression. As a consequence, to more fully understand the functions of DEGs and analyze their regulatory mechanisms, we predicted possible TF genes from among the 435 common DEGs shared in all comparisons using the plant TF database PlantTFDB (Jin et al., 2017). In total, 40 genes were identified, including 33 upregulated genes and seven downregulated genes, and they were grouped into 11 different TF families (Figure 5; Figure 6, Table S6). Among them, the ERF family was the most abundant family (13 genes). The remaining top 4 TF families were bHLH (seven genes), NAC (five genes), C2H2 (four genes), and B3 (three genes).

## Discussion

In the natural environment, plants are constantly threatened by herbivorous insects. For Chinese chestnut, damage from spruce spider mite is a primary challenge restricting its output and quality, and generally leads to a serious commercial loss. In previous work, we developed several shade-tolerant foxtail millet varieties for interplanting with chestnut trees, and found that it improved the quality of chestnut fruits by attracting spruce spider mite to feed on their leaves. This strategy could become a new, effective, and environmentally friendly, pest-management option for chestnut tree.

Nutrients of host plants, such as proteins and carbohydrates, are essential sources of energy for the growth and development of herbivorous insects. CP content in leaves affects the lifespan, development duration, and egg laying outcomes of mites, and in certain plant species the degree of leaf damage by insects is higher for leaves with higher CP levels (Wu et al., 1995; Fan et al., 2008; Wei et al., 2009; Yang et al., 2014; Sonalkar, 2020). The foxtail millet varieties we previously bred for interplanting with chestnut tree all had high CP levels in leaves, which may be one major reason why they attracted the spider mites. In present study, four foxtail millet varieties with significant differences in leaf CP content were used to identify genes related to CP levels *via* transcriptome sequencing technology. We successfully identified 435 DEGs, including 337 upregulated DEGs and 98 downregulated DEGs in varieties with high CP concentrations. Our findings provide a precious resource for research on the mechanisms that control the accumulation of CP, and offer a chance to effectively control spruce spider mite outbreaks among chestnut trees.

Protein is an important product of nitrogen metabolism, and therefore CP levels are closely associated with nitrogen metabolism. For example, an increase in CP levels of vegetable leaves is correlated with an increase in N uptake (Mhlontlo et al., 2007). Most plants assimilate inorganic and organic forms of nitrogen, including nitrate and ammonium (Temple et al., 1998). Metabolism normally begins with nitrate being converted into nitrite in the presence of nitrate reductase, and then the nitrite is reduced to form ammonia by nitrite reductase. Following its formation, ammonia is assimilated by a number of pathways, including reductive amination and transpiration, to yield a variety of amino acids (Mokhele et al., 2012; Kishorekumar et al., 2020). In the current study, two DEGs, SETIT_014128 mg and SETIT_014124mg, were enriched in the nitrogen metabolism pathway and may code for carbonic anhydrase (CA). The reversible conversion of carbon dioxide to bicarbonate (CO_2_+H_2_O ⇔ HCO_3_^−^ + H^+^) is catalyzed by this enzyme in the leaves of C_4_ plants (Ludwig, 2016). Cyanate in plants, which is derived from endogens cyanide or comes from the environment, is decomposed into carbon dioxide and ammonia (CNO^−^ + HCO_3_^−^+2H^+^ ⇔ NH_3_+2CO_2_), and produced ammonia enters into biosynthesis pathway of amino acids. This reaction is bicarbonate-dependent (Johnson and Anderson, 1987; Kozliak et al., 1995; Ebbs, 2004; Yu et al., 2019). Thus, SETIT_014128 mg and SETIT_014124 mg may promote nitrogen metabolism and subsequent biosynthesis of proteins by upregulating their expression level and the activity of CA. In addition, in the first step of C_4_ photosynthesis, phosphoenolpyruvate carboxylase (PEPC) uses bicarbonate as a substrate, whose generation from carbon dioxide is catalyzed by CA (Ludwig, 2016); the decrease in CA accumulation and CA activity plays a considerable role in protein accumulation of PEPC (Chatterjee et al., 2021). In addition to having a significant impact on carbon and nitrogen metabolism, PEPC also influences protein synthesis by promoting the distribution of the carbon skeleton to amino acid production (Shinano et al., 2006). One study found that applying the nanobiomaterial ‘nanochitin whisker’ primarily facilitated the accumulation of nitrogen and translocation more than carbon metabolism and enhanced CP concentration in grain by stimulating PEPC activity and reducing the ratios of sucrose phosphate synthase (SPS) to PEPC in flag leaves and spikes of wheat during the anthesis and grain-filling stages (Cheng et al., 2019), revealing the role of PEPC in the coordination of carbon and nitrogen flow to amino acids and carbohydrates. These findings further support that SETIT_014128 mg and SETIT_014124 mg may be hub genes associated with leaf CP concentration in foxtail millet, and can be used to develop high-CP varieties by genetic manipulation; however, their detailed functions need to be further clarified.

In eukaryotes, the ubiquitin-proteasome system (UPS) is responsible for the majority of cytosolic protein breakdown, accomplished through the degradation of the substrate protein by ATP-dependent 26S proteasome after covalent attachment of many Ub molecules to the substrate protein (Ciechanover et al., 2000; Nandi et al., 2006). Binding of Ub to target proteins includes the following steps. First, the C-terminal Gly of Ub is activated and transferred to a carrier E2 protein (Ub-conjugating enzyme) by the Ub-activating enzyme E1. Then with the aid of E3 (a Ub-protein ligase enzyme), the E2 protein tags Ub to the target protein. Various E3 ligases within the UPS recognize substrates carrying diverse degradation signals, giving the system high specificity and selectivity. In this study, we identified two DEGs that encode Ub-conjugating enzyme E2 J2 (UBE2J2) and E3 Ub-protein ligase SIAH1, namely, SETIT_025290 mg and SETIT_004756mg, respectively. The UBE2J2 protein is a Ub-conjugating enzyme which, in association with Ub ligases including TEB4, PARKIN, CHIP, and cIAP1, promotes proteasomal degradation in mammals and responds to proteotoxic stress (Imai et al., 2002; Lenk et al., 2002; Kim et al., 2003; Wu et al., 2005; Choi et al., 2010; Claessen et al., 2010; Burr et al., 2011). Nevertheless, no UBE2J2 proteins have been characterized in plants to date. The seven in absentia (SINA)/seven in absentia homolog (SIAH) family proteins are E3 Ub-protein ligases with a RING domain and are highly conserved from plants to mammals (Polekhina et al., 2002). This superfamily was first characterized in *Drosophila melanogaster* and plays a crucial role in the regulation of development and proteasome-mediated protein degradation (Hu and Fearon Eric, 1999). To date, 18 SINA proteins homologous to the *Drosophila* SINA protein have been defined in *Arabidopsis*, among which SINAT5 targets the noapical meristem/cup-shaped cotyledon 1 (NAC1) TF and late elongated hypocotyl (LHY) for degradation and plays a role in various developmental processes in plants (Xie et al., 2002; Park et al., 2010). Therefore, SETIT_025290 mg and SETIT_004756 mg may be involved in specific ubiquitination and subsequent proteolysis of proteins, and have an impact on leaf CP content in foxtail millet by regulating protein levels and the stability of related proteins. However, their biological functions and regulation mechanisms remain to be elucidated in future work.

TFs regulate a variety of biological processes, such as plant metabolism, growth, and development, by binding to the promoters (or intragenic regions) of target genes (Kaufmann et al., 2018; Burgess et al., 2019). Many TFs have been found to control nitrogen assimilation. Recently, Wei et al. reported that overexpression of the *dehydration-responsive element-binding protein 1C* (*OsDREB1C*) gene in rice (*Oryza sativa*), a member of the AP2/ERF TF family, improved nitrogen content, nitrogen use efficiency (NUE), and protein abundance in leaves by elevating nitrogen uptake and transport activity (Wei et al., 2022). Furthermore, a few TFs affect protein synthesis in plants. For example, overexpressing the MYB family TF *TaODORANT1* in common wheat (*Triticum aestivum*) and its homolog in *Triticum urartu* reduces the transcription levels of seed storage protein (SSP) genes and total SSP levels of mature grains, revealing an inhibition effect on SSP synthesis (Luo et al., 2021). *OPAQUE 11* (*O11*) encodes a seed-specific bHLH TF in maize, and the protein levels of loss-of-function mutant *o11* endosperm per kernel are significantly decreased compared to wild type (Feng et al., 2018), revealing regulatory role in protein accumulation. In addition, several NAC TFs have also been reported to regulate protein accumulation and content in grains of wheat, rice, and maize (*Zea mays*) (Uauy et al., 2006; Liang et al., 2014; Zhang et al., 2019; Wang et al., 2020). An endosperm-specific NAC TF, TaNAC019, modulates SSP accumulation in wheat seeds (Gao et al., 2021). It directly binds to the promoters of *high-molecular-weight glutenin* (*HMW-GS*) genes and activates their expression, and triple knock-out mutants of *TaNAC019* homologs have lower gluten levels. In the current research, 40 DEGs were identified as TF genes, containing several members of the bHLH, MYB, NAC, and especially ERF gene families (Figure 5). It can be speculated that these genes may function as TFs and improve CP accumulation in leaves of foxtail millet by modulating nitrogen assimilation and protein synthesis. However, the detailed underlying molecular mechanisms involved require further research, and will be a focus of ours in the future.

## Conclusion

Transcriptome analysis of genes in foxtail millet associated with CP accumulation in leaves was performed and 435 DEGs were identified. Several DEGs related to nitrogen metabolism and ubiquitin-mediated proteolysis pathways were characterized. We also predicted the TFs that participate in the control of CP levels. These findings provide a resource that clarifies the accumulation and control mechanisms of CP levels in leaves. This information can be used to develop foxtail millet varieties with high CP concentrations for interplanting with chestnut, to improve production of this important crop.

## Data Availability

The original contributions presented in the study are publicly available. This data can be found here: https://www.ncbi.nlm.nih.gov/bioproject/PRJNA772942.

## References

[B1] Adua M. (1999). The sweet chestnut throughout history from the Miocene to the third millennium. Acta Hortic. 494, 29–36. 10.17660/actahortic.1999.494.2

[B2] Belete T. (2018). Defense mechanisms of plants to insect pests: From morphological to biochemical approach. Trends Tech. Sci. Res. 2 (2), 30–38. 10.19080/ttsr.2018.02.555584

[B3] Burgess S. J. Reyna-Llorens I. Stevenson S. R. Singh P. Jaeger K. Hibberd J. M. (2019). Genome-wide transcription factor binding in leaves from C3 and C4 grasses. Plant Cell. 31 (10), 2297–2314. 10.1105/tpc.19.00078 31427470 PMC6790085

[B4] Burr M. L. Cano F. Svobodova S. Boyle L. H. Boname J. M. Lehner P. J. (2011). HRD1 and UBE2J1 target misfolded MHC class I heavy chains for endoplasmic reticulum-associated degradation. Proc. Natl. Acad. Sci. U. S. A. 108 (5), 2034–2039. 10.1073/pnas.1016229108 21245296 PMC3033308

[B5] Chatterjee J. Coe R. A. Acebron K. Thakur V. Yennamalli R. M. Danila F. (2021). A low CO_2_-responsive mutant of *Setaria viridis* reveals that reduced carbonic anhydrase limits C4 photosynthesis. J. Exp. Bot. 72 (8), 3122–3136. 10.1093/jxb/erab039 33528493 PMC8023212

[B6] Chen C. Chen H. Zhang Y. Thomas H. R. Frank M. H. He Y. (2020). TBtools: An integrative toolkit developed for interactive analyses of big biological data. Mol. Plant 13 (8), 1194–1202. 10.1016/j.molp.2020.06.009 32585190

[B7] Cheng Y. Wang Y. Han Y. Li D. Zhang Z. Zhu X. (2019). The stimulatory effects of nanochitin whisker on carbon and nitrogen metabolism and on the enhancement of grain yield and crude protein of winter wheat. Molecules 24 (9), 1752. 10.3390/molecules24091752 31064118 PMC6539796

[B8] Choi Y. L. Soda M. Yamashita Y. Ueno T. Takashima J. Nakajima T. (2010). EML4-ALK mutations in lung cancer that confer resistance to ALK inhibitors. New Engl. J. Med. 363 (18), 1734–1739. 10.1056/NEJMoa1007478 20979473

[B9] Ciechanover A. Orian A. Schwartz A. L. (2000). Ubiquitin-mediated proteolysis: Biological regulation via destruction. Bioessays 22 (5), 442–451. 10.1002/(SICI)1521-1878(200005)22:5<442:AID-BIES6>3.0.CO;2-Q 10797484

[B10] Claessen J. H. Mueller B. Spooner E. Pivorunas V. L. Ploegh H. L. (2010). The transmembrane segment of a tail-anchored protein determines its degradative fate through dislocation from the endoplasmic reticulum. J. Biol. Chem. 285 (27), 20732–20739. 10.1074/jbc.M110.120766 20435896 PMC2898349

[B11] Ebbs S. (2004). Biological degradation of cyanide compounds. Curr. Opin. Biotechnol. 15 (3), 231–236. 10.1016/j.copbio.2004.03.006 15193331

[B12] Fan Y. Jiang X. Hao J. Liu Z. Wang H. Wu Y. (2008). Correlation analysis between damage of the grape leafhopper and the chemicals of grape varieties in Xinjiang. Acta Agric. Boreali-occidentalis Sin. 17 (1), 70–73. 10.3969/j.issn.1004-1389.2008.01.016

[B13] Feng F. Qi W. Lv Y. Yan S. Xu L. Yang W. (2018). OPAQUE11 is a central hub of the regulatory network for maize endosperm development and nutrient metabolism. Plant Cell. 30 (2), 375–396. 10.1105/tpc.17.00616 29436476 PMC5868688

[B14] Gao Y. An K. Guo W. Chen Y. Zhang R. Zhang X. (2021). The endosperm-specific transcription factor TaNAC019 regulates glutenin and starch accumulation and its elite allele improves wheat grain quality. Plant Cell. 33 (3), 603–622. 10.1093/plcell/koaa040 33955492 PMC8136912

[B15] Gokce M. P. Karagoz M. Faraji F. Cakmak I. (2020). Mite species composition and their population densities on chestnut trees in Turkey. Int. J. Acarol. 46 (4), 247–253. 10.1080/01647954.2020.1752796

[B16] Hershko A. (1998). “The ubiquitin system,” in Ubiquitin and the biology of the cell. Editors Peters J-M. Harris J. R. Finley D. (Boston, MA: Springer US), 1–17.

[B17] Hu G. Fearon Eric R. (1999). Siah-1 N-terminal RING domain is required for proteolysis function, and C-terminal sequences regulate oligomerization and binding to target proteins. Mol. Cell. Biol. 19 (1), 724–732. 10.1128/mcb.19.1.724 9858595 PMC83929

[B18] Imai Y. Soda M. Hatakeyama S. Akagi T. Hashikawa T. Nakayama K-I. (2002). CHIP is associated with Parkin, a gene responsible for familial Parkinson's disease, and enhances its ubiquitin ligase activity. Mol. Cell. 10 (1), 55–67. 10.1016/s1097-2765(02)00583-x 12150907

[B19] Iseli C. Jngeneel C. V. Bucher P. (1999). ESTScan: A program for detecting, evaluating, and reconstructing potential coding regions in EST sequences. Proc. Int. Conf. Intell. Syst. Mol. Biol. 99, 138–148. 10786296

[B20] Jiang N. Fan X. L. Tian C. M. (2019). Identification and pathogenicity of Cryphonectriaceae species associated with chestnut canker in China. Plant Pathol. 68 (6), 1132–1145. 10.1111/ppa.13033

[B21] Jin J. Tian F. Yang D. C. Meng Y. Q. Kong L. Luo J. (2017). PlantTFDB 4.0: Toward a central hub for transcription factors and regulatory interactions in plants. Nucleic Acids Res. 45 (D1), D1040–D1045. 10.1093/nar/gkw982 27924042 PMC5210657

[B22] Johnson W. V. Anderson P. M. (1987). Bicarbonate is a recycling substrate for cyanase. J. Biol. Chem. 262 (19), 9021–9025. 10.1016/s0021-9258(18)48040-4 3110153

[B23] Kaufmann K. Airoldi C. A. (2018). “Master regulatory transcription factors in plant development: A blooming perspective,” in Plant transcription factors: Methods and protocols. Editor Yamaguchi N. (New York, NY: Springer New York), 3–22. 10.1007/978-1-4939-8657-6_1 30043361

[B24] Kim B. W. Zavacki A. M. Curcio-Morelli C. Dentice M. Harney J. W. Larsen P. R. (2003). Endoplasmic reticulum-associated degradation of the human type 2 iodothyronine deiodinase (D2) is mediated via an association between mammalian UBC7 and the carboxyl region of D2. Mol. Endocrinol. 17 (12), 2603–2612. 10.1210/me.2003-0082 12933904

[B25] Kishorekumar R. Bulle M. Wany A. Gupta K. J. (2020). An overview of important enzymes involved in nitrogen assimilation of plants. Methods Mol. Biol. 2057, 1–13. 10.1007/978-1-4939-9790-9_1 31595465

[B26] Kjeldahl J. (1883). Neue Methode zur Bestimmung des Stickstoffs in organischen Körpern. Z Anal. Chem. 22 (1), 366–382. 10.1007/bf01338151

[B27] Kozliak E. I. Fuchs J. A. Guilloton M. B. Anderson P. M. (1995). Role of bicarbonate/CO_2_ in the inhibition of *Escherichia coli* growth by cyanate. J. Bacteriol. 177 (11), 3213–3219. 10.1128/jb.177.11.3213-3219.1995 7768821 PMC177013

[B28] Lenk U. Yu H. Walter J. Gelman M. S. Hartmann E. Kopito R. R. (2002). A role for mammalian Ubc6 homologues in ER-associated protein degradation. J. Cell. Sci. 115 (14), 3007–3014. 10.1242/jcs.115.14.3007 12082160

[B29] Li S. Cui Y. Liu D. Zhao Z. Zhang J. Liu Z. (2022). Transcriptome analysis and characterization of genes associated to leaf tannin content in foxtail millet [*Setaria italica* (L.) P. Beauv.]. BMC Genom 23 (1), 512. 10.1186/s12864-022-08746-8 PMC9284691 35836120

[B30] Li L. Jin D. (2005). Main factors on interrelationship between phytophagous mites and parasite plants. Guizhou Agr. Sci. 33 (4), 95–97. 10.3969/j.issn.1001-3601.2005.04.045

[B31] Li W. Li S. (2018). Advances in insect resistance of forest trees. J. Southwest For. Univ. 38 (5), 180–190.

[B32] Liang C. Wang Y. Zhu Y. Tang J. Hu B. Liu L. (2014). OsNAP connects abscisic acid and leaf senescence by fine-tuning abscisic acid biosynthesis and directly targeting senescence-associated genes in rice. Proc. Natl. Acad. Sci. U. S. A. 111 (27), 10013–10018. 10.1073/pnas.1321568111 24951508 PMC4103337

[B33] Liu D. Cui Y. Zhao Z. Li S. Liang D. Wang C. (2021). Genome-wide identification and characterization of the BES/BZR gene family in wheat and foxtail millet. BMC Genom 22 (1), 682. 10.1186/s12864-021-08002-5 PMC8456565 34548036

[B34] Liu D. Cui Y. Zhao Z. Zhang J. Li S. Liu Z. (2022). Transcriptome analysis and mining of genes related to shade tolerance in foxtail millet (*Setaria italica* (L.) P. Beauv.). Roy. Soc. Open Sci. 9 (10), 220953. 10.1098/rsos.220953 36249327 PMC9532984

[B35] Ludwig M. (2016). Evolution of carbonic anhydrase in C4 plants. Curr. Opin. Plant Biol. 31, 16–22. 10.1016/j.pbi.2016.03.003 27016649

[B36] Luo G. Shen L. Song Y. Yu K. Ji J. Zhang C. (2021). The MYB family transcription factor TuODORANT1 from *Triticum urartu* and the homolog TaODORANT1 from *Triticum aestivum* inhibit seed storage protein synthesis in wheat. Plant Biotechnol. J. 19 (9), 1863–1877. 10.1111/pbi.13604 33949074 PMC8428827

[B37] Mhlontlo S. Muchaonyerwa P. Mnkeni P. (2007). Effects of sheep kraal manure on growth, dry matter yield and leaf nutrient composition of a local <i&gt;Amaranthus&lt;/i&gt; accession in the central region of the Eastern Cape Province, South Africa. Water sa. 33 (3), 363–368. 10.4314/wsa.v33i3.180597

[B38] Mokhele B. Zhan X. Yang G. Zhang X. (2012). Review: Nitrogen assimilation in crop plants and its affecting factors. Can. J. Plant Sci. 92 (3), 399–405. 10.4141/cjps2011-135

[B39] Nandi D. Tahiliani P. Kumar A. Chandu D. (2006). The ubiquitin-proteasome system. J. Biosci. 31 (1), 137–155. 10.1007/BF02705243 16595883

[B40] Park B. S. Eo H. J. Jang I. C. Kang H. G. Song J. T. Seo H. S. (2010). Ubiquitination of LHY by SINAT5 regulates flowering time and is inhibited by DET1. Biochem. Biophys. Res. Commun. 398 (2), 242–246. 10.1016/j.bbrc.2010.06.067 20599732

[B41] Polekhina G. House C. M. Traficante N. Mackay J. P. Relaix F. Sassoon D. A. (2002). Siah ubiquitin ligase is structurally related to TRAF and modulates TNF-alpha signaling. Nat. Struct. Biol. 9 (1), 68–75. 10.1038/nsb743 11742346

[B42] Rizwan M. Abro S. Asif M. U. Hameed A. Mahboob W. Deho Z. A. (2021). Evaluation of cotton germplasm for morphological and biochemical host plant resistance traits against sucking insect pests complex. J. Cotton Res. 4 (1), 18–8. 10.1186/s42397-021-00093-5

[B43] Robinson M. D. McCarthy D. J. Smyth G. K. (2009). edgeR: a Bioconductor package for differential expression analysis of digital gene expression data. Bioinformatics 26 (1), 139–140. 10.1093/bioinformatics/btp616 19910308 PMC2796818

[B44] Shinano T. Nakajima K. Wasaki J. Mori H. Zheng T. Osaki M. (2006). Developmental regulation of photosynthate distribution in leaves of rice. Photosynthetica 44 (1), 1–10. 10.1007/s11099-005-0151-6

[B45] Sonalkar V. U. (2020). Biochemicals in cotton hybrids and varieties and their correlation with sucking insect pests. Inte J. Curr. Microbiol. Appl. Sci. 9 (1), 1172–1183. 10.20546/ijcmas.2020.901.132

[B46] Tanaka K. Kotobuki K. (1992). Studies on peeling characteristics of Japanese chestnut (*Castanea crenata* Sieb. et Zucc), Chinese chestnut (*C. mollissima* BL.) and their hybrids. Acta Hortic. 317, 175–180. 10.17660/actahortic.1992.317.20

[B47] Temple S. J. Vance C. P. Stephen Gantt J. (1998). Glutamate synthase and nitrogen assimilation. Trends Plant Sci. 3 (2), 51–56. 10.1016/s1360-1385(97)01159-x

[B48] Trapnell C. Williams B. A. Pertea G. Mortazavi A. Kwan G. van Baren M. J. (2010). Transcript assembly and quantification by RNA-Seq reveals unannotated transcripts and isoform switching during cell differentiation. Nat. Biotechnol. 28 (5), 511–515. 10.1038/nbt.1621 20436464 PMC3146043

[B49] Uauy C. Distelfeld A. Fahima T. Blechl A. Dubcovsky J. (2006). A NAC gene regulating senescence improves grain protein, zinc, and iron content in wheat. Science 314, 1298–1301. 10.1126/science.1133649 17124321 PMC4737439

[B50] Wang Y. Wang Y. Xu J. (1989). Seasonal occurrence and control of Spruce spider mite, *Oligonychus ununguis* (Jacobi) on chestnut trees. Acta Agric. boreali-sinica. 4 (4), 98–104. 10.3321/j.issn:1000-7091.1989.04.019

[B51] Wang B. Jiang Y. Zhang F. Ren W. (1991). Two pestcides for prevention of spruce spider mite on Chinese chestnut. For. Pest Dis. (4), 52.

[B52] Wang X. Wen X. Lu C. (2010). Research advance in controlling insect pests on Castanea mollissima in China. J. Hebei Normal Univ. Science&Technology 24 (1), 39–43. 10.3969/j.issn.1672-7983.2010.01.011

[B53] Wang J. Chen Z. Zhang Q. Meng S. Wei C. (2020). The NAC transcription factors OsNAC20 and OsNAC26 regulate starch and storage protein synthesis. Plant Physiol. 184 (4), 1775–1791. 10.1104/pp.20.00984 32989010 PMC7723083

[B54] War A. R. Paulraj M. G. Ahmad T. Buhroo A. A. Hussain B. Ignacimuthu S. (2012). Mechanisms of plant defense against insect herbivores. Plant Signal Behav. 7 (10), 1306–1320. 10.4161/psb.21663 22895106 PMC3493419

[B55] Wei W. Qin W. Wei M. Lu L. Tang X. He H. (2009). Resistances of different cassava varieties to Tetranychus urticae. J. South. Agric. 40 (5), 504–506. 10.3969/j.issn.2095-1191.2009.05.013

[B56] Wei S. Li X. Lu Z. Zhang H. Ye X. Zhou Y. (2022). A transcriptional regulator that boosts grain yields and shortens the growth duration of rice. Science 377 (6604), eabi8455. 10.1126/science.abi8455 35862527

[B57] Wu Y. Liu Q. Liu H. Wu W. (1995). Induction of nitric oxide synthase and motoneuron death in newborn and early postnatal rats following spinal root avulsion. Acta Gossypii Sin. 7 (2), 109–112. 10.1016/0304-3940(95)11741-e 7478189

[B58] Wu C. J. Conze D. B. Li X. Ying S. X. Hanover J. A. Ashwell J. D. (2005). TNF-alpha induced c-IAP1/TRAF2 complex translocation to a Ubc6-containing compartment and TRAF2 ubiquitination. EMBO J. 24 (10), 1886–1898. 10.1038/sj.emboj.7600649 15861135 PMC1142588

[B59] Xie Q. Guo H-S. Dallman G. Fang S. Weissman A. M. Chua N-H. (2002). SINAT5 promotes ubiquitin-related degradation of NAC1 to attenuate auxin signals. Nature 419 (6903), 167–170. 10.1038/nature00998 12226665

[B60] Xie C. Mao X. Huang J. Ding Y. Wu J. Dong S. (2011). Kobas 2.0: A web server for annotation and identification of enriched pathways and diseases. Nucleic Acids Res. 39, W316–W322. 10.1093/nar/gkr483 21715386 PMC3125809

[B61] Yang N. Wu S. Shen L. Zhang S. Yang B. (2014). A review on plant resistance to insect pests. Chin. J. Trop. Agric. 34 (9), 61–68+89. 10.3969/j.issn.1009-2196.2014.09.014

[B62] Yu G. Li F. Qin Y. Bo X. Wu Y. Wang S. (2010). GOSemSim: an R package for measuring semantic similarity among GO terms and gene products. Bioinformatics 26 (7), 976–978. 10.1093/bioinformatics/btq064 20179076

[B63] Yu X. Z. Lei S. Y. Lin Y. J. Zhang Q. (2019). Interaction of cyanate uptake by rice seedlings with nitrate assimilation: Gene expression analysis. Environ. Sci. Pollut. Res. Int. 26 (20), 20208–20218. 10.1007/s11356-019-05407-4 31098903

[B64] Zhang Z. Dong J. Ji C. Wu Y. Messing J. (2019). NAC-type transcription factors regulate accumulation of starch and protein in maize seeds. Proc. Natl. Acad. Sci. U. S. A. 116 (23), 11223–11228. 10.1073/pnas.1904995116 31110006 PMC6561305

[B65] Zhao H. Fan R. (2019). Occurrence and control methods of red spiders of chestnut. Mod. Rural Sci. Technol. 9, 34. 10.3969/j.issn.1674-5329.2019.09.028

[B66] Zhou H. Zhao J. Cai J. Patil S. B. (2017). UBIQUITIN-SPECIFIC PROTEASES function in plant development and stress responses. Plant Mol. Biol. 94 (6), 565–576. 10.1007/s11103-017-0633-5 28695315

