# Supplementary material for: Transcriptome analysis and identification of genes associated with leaf crude protein content in foxtail millet [Setaria italica (L.) P. Beauv.]
Source: Front Genet. 2023 Jan 20;14:1122212. doi: 10.3389/fgene.2023.1122212 (PMC9895776; doi:10.3389/fgene.2023.1122212)
Supplement: Supplementary file 1 [file DataSheet1.ZIP › Supplementary material/Supplementary_Material.docx]

Supplementary Material

Transcriptome analysis and identification of genes associated with leaf crude protein content in foxtail millet [*Setaria italica* (L.) P. Beauv.]

Yanjiao Cui^1†^, Dan Liu^2^^†^, Zilong Zhao^1^, Jing Zhang^1^, Suying Li^1*^, Zhengli Liu^1*^

*** Correspondence:** Zhengli Liu: [liuzhengli65@126.com](mailto:liuzhengli65@126.com), Suying Li: [lisuying65@126.com](mailto:lisuying65@126.com)

# Supplementary Materials and Methods

**Whole transcriptome library construction and high-throughput sequencing**

To mine genes related to leaf CP content, the middle parts of the first, second, and third leaves from the top of plants in the booting stage were sampled from the four foxtail millet hybrid varieties 57295, 51950, 12950, and 1121. Three biological replicates were performed for each sample, and three standard plants were selected for each replicate. Sampled leaves from each variety were mixed and flash frozen in liquid nitrogen for total RNA isolation.

Total RNA isolation, preparation of whole transcriptome libraries, and deep sequencing were performed by Berry Genomics Co., Ltd. (Beijing, China). In all, 2 μg high-quality RNA per sample was used to construct cDNA libraries using the VAHTS mRNA-seq v2 Library Prep Kit for Illumina following the manufacturer’s recommendations and index codes were added to attribute sequences to each sample. Briefly, mRNA was purified from total RNA using poly-T oligo-attached magnetic beads. Fragmentation was carried out using fragmentation buffer. First-strand cDNA was synthesized and second-strand cDNA synthesis was subsequently performed. Remaining overhangs were converted into blunt ends. After adenylation of 3’ ends of DNA fragments, adaptors with hairpin loop structures were ligated. Then PCR was performed. Finally, Qubit HS quantification, Agilent 2100 Bioanalyzer/Fragment Analyzer 5300 quality control, the final library size of about 350 bp. The libraries were sequenced on an Illumina NovaSeq platform that generated paired end reads (raw data) of 150 bp.

**Transcriptome assembly**

The raw data (raw reads) in fastq format were processed through primary quality control. In this step, clean data (clean reads) were obtained by removing read pairs that contained N more than 3 or the proportion of base with quality value below 5 is more than 20%, in any end, or adapter sequence was founded. All downstream analyses were based on clean data of high quality. Efficient alignment between the clean reads and the foxtail millet reference genome (Setaria_italica_v2.0, http://plants.ensembl.org/Setaria_italica/Info/Index) was performed using HISAT2 version 2.0.6 (1), and the mapped reads were assembled using Cufflinks version 2.2.1 (2).

**qRT-PCR validation**

Total RNA was extracted using the RNApre Pure Plant Kit (Tiangen, China), and cDNA was synthesized with a Revert Aid First Strand cDNA Synthesis Kit (Thermo Fisher Scientific, USA) according to the manufacturer’s instructions. qRT-PCR utilized SuperReal PreMix Plus (SYBR Green, TIANGEN, China) on a QuantStudio 6 Flex Real-Time PCR system (Applied Biosystems, USA) following the manufacturer’s instructions. The PCR thermal profile employed the following parameters: 95°C for 15 s, 40 cycles at 95°C for 10 s, and 60°C for 31 s. Then the relative expression level of each gene was calculated based on the equation 2^−ΔΔCT^ (3). The *SiACTIN* gene was used as an internal control. All data were generated by averaging three independent replicates. All primers are listed in Table S2.

# Supplementary Figures and Tables

**Table S1.** The crude protein content in leaves of different foxtail millet varieties.

| Variety | CP content of the leaves (%) | | | Average (%) | Percentage of CP content changes compared to JG32 (%) | Percentage of CP content changes compared to 57295 (%) |
| --- | --- | --- | --- | --- | --- | --- |
| 1121 | 5.09 | 5.22 | 5.03 | 5.11^a^ | 14.83 | 37.37 |
| 51950 | 4.92 | 4.87 | 4.79 | 4.86^ab^ | 9.21 | 30.65 |
| 12950 | 4.93 | 4.81 | 4.73 | 4.82^b^ | 8.31 | 29.57 |
| JG32 | 4.51 | 4.19 | 4.66 | 4.45^c^ | - | 19.62 |
| 57295 | 3.92 | 3.67 | 3.56 | 3.72^d^ | 16.40 | - |

The letters a-d denote within rows with the same superscript are not significantly different (ANOVA, P>0.05, α=0.05).

**Table S2.** Primer sequences used for quantitative real-time PCR.

| Gene name | Primer sequence (5’-3’) | |
| --- | --- | --- |
|  | Forward | Reverse |
| SETIT_031738mg | CATGGCTCTGCTTGTGCAAA | CTGCTGCGTTGTGGGTTCT |
| SETIT_020998mg | GGCCAAAGATCGCCCATAT | GGGATCCGAAGCGCAATT |
| SETIT_025437mg | TTCTCATTCTTTTCCCTTTTCTGTCT | CACGAGAACCCTCCCGATT |
| SETIT_009199mg | GGGTGCACTTTGGTGCTTATTT | GGGCAAGTCCCGCATCT |
| SETIT_009896mg | GCCCTGCTGAGAGCCATAAG | CGACCACGCGAAGTATGCT |
| SETIT_012108mg | CCGTCGCCATGAACCTCTA | GCGCGGAGCTGTTGAAGTAG |
| SETIT_038934mg | GGCGGTCTACCGACGTACTG | CGGCGGACACATTTTTTCAT |
| SETIT_0403631mg | AGATGTGTGCCGGATCTACATG | CTTCGCTGCTGGCTTGCT |
| SiACTIN | GGATACTCTTTCACCACCTC | ACCTCAGGGCACCTAAAC |

**Table S3.** FPKM values of genes identified in this study. The suffixes -1, -2, -3 indicate three biological replicates for each sample.

**Table S4.** GO annotation of the differentially expressed genes shared in all comparisons.

**Table S5.** KEGG pathway annotation and classification of the differentially expressed genes shared in all comparisons.

**Table S6.** Differentially expressed transcription factor genes. Up indicates gene is upregulated and down indicates gene is downregulated.

| TF family | Gene numbers | GeneID | Change of expression level |
| --- | --- | --- | --- |
| B3 | 3 | SETIT_0100262mg | down |
|  |  | SETIT_010155mg | down |
|  |  | SETIT_012530mg | down |
| bHLH | 7 | SETIT_002847mg | up |
|  |  | SETIT_010413mg | up |
|  |  | SETIT_010812mg | up |
|  |  | SETIT_014241mg | up |
|  |  | SETIT_024550mg | down |
|  |  | SETIT_026212mg | up |
|  |  | SETIT_030966mg | up |
| C2H2 | 4 | SETIT_037251mg | up |
|  |  | SETIT_037672mg | up |
|  |  | SETIT_037937mg | up |
|  |  | SETIT_039857mg | up |
| ERF | 13 | SETIT_002714mg | down |
|  |  | SETIT_007199mg | up |
|  |  | SETIT_010228mg | up |
|  |  | SETIT_014469mg | up |
|  |  | SETIT_014474mg | up |
|  |  | SETIT_018262mg | up |
|  |  | SETIT_018550mg | up |
|  |  | SETIT_019997mg | up |
|  |  | SETIT_023330mg | up |
|  |  | SETIT_024861mg | down |
|  |  | SETIT_030869mg | up |
|  |  | SETIT_030998mg | up |
|  |  | SETIT_031001mg | up |
| GRAS | 1 | SETIT_040237mg | up |
| HD-ZIP | 1 | SETIT_037255mg | up |
| M-type_MADS | 2 | SETIT_005133mg | down |
|  |  | SETIT_008439mg | up |
| MYB | 2 | SETIT_026574mg | up |
|  |  | SETIT_030273mg | up |
| NAC | 5 | SETIT_004307mg | up |
|  |  | SETIT_006975mg | up |
|  |  | SETIT_010553mg | up |
|  |  | SETIT_032755mg | up |
|  |  | SETIT_036695mg | up |
| Nin-like | 1 | SETIT_016430mg | up |
| WRKY | 1 | SETIT_017443mg | up |

**References**

1.Kim D, Langmead B, Salzberg SL. HISAT: a fast spliced aligner with low memory requirements. Nat Methods. 2015;12(4):357-60.

2.Trapnell C, Williams BA, Pertea G, Mortazavi A, Kwan G, van Baren MJ, et al. Transcript assembly and quantification by RNA-Seq reveals unannotated transcripts and isoform switching during cell differentiation. Nat Biotechnol. 2010;28(5):511-5.

3.Schmittgen TD, Livak KJ. Analyzing real-time PCR data by the comparative CT method. Natu Protoc. 2008;3(6):1101-8.
